# Ubiquitin-proteasome pathway in skeletal muscle atrophy

**DOI:** 10.3389/fphys.2023.1289537

**Published:** 2023-11-17

**Authors:** XiangSheng Pang, Peng Zhang, XiaoPing Chen, WenMing Liu

**Affiliations:** ^1^ Department of Physical Education, College of Education, Zhejiang University, Hangzhou, Zhejiang, China; ^2^ National Key Laboratory of Human Factors Engineering, China Astronaut Research and Training Center, Beijing, China; ^3^ State Key Laboratory of Space Medicine Fundamentals and Application, China Astronaut Research and Training Center, Beijing, China

**Keywords:** skeletal muscle atrophy, ubiquitin-proteasom, muscle disuse, sarcopenia, cachexia

## Abstract

Skeletal muscles underpin myriad human activities, maintaining an intricate balance between protein synthesis and degradation crucial to muscle mass preservation. Historically, disruptions in this balance—where degradation overshadows synthesis—have marked the onset of muscle atrophy, a condition diminishing life quality and, in grave instances, imperiling life itself. While multiple protein degradation pathways exist—including the autophagy-lysosome, calcium-dependent calpain, and cysteine aspartate protease systems—the ubiquitin-proteasome pathway emerges as an especially cardinal avenue for intracellular protein degradation, wielding pronounced influence over the muscle atrophy trajectory. This paper ventures a panoramic view of predominant muscle atrophy types, accentuating the ubiquitin-proteasome pathway’s role therein. Furthermore, by drawing from recent scholarly advancements, we draw associations between the ubiquitin-proteasome pathway and specific pathological conditions linked to muscle atrophy. Our exploration seeks to shed light on the ubiquitin-proteasome pathway’s significance in skeletal muscle dynamics, aiming to pave the way for innovative therapeutic strategies against muscle atrophy and affiliated muscle disorders.

## 1 Introduction

Skeletal muscles, constituting over 40% of body weight in healthy adults, stand as the body’s most prolific tissue. Beyond facilitating movement, these muscles function as pivotal protein stores, orchestrating metabolic processes and ensuring glucose equilibrium. Intrinsically designed to swiftly adjust to both external and internal shifts, skeletal muscle’s adaptability becomes fundamental to tissue vitality and overall survival. Central to this functionality is the preservation of muscle mass, critically underpinned by the equilibrium between protein synthesis and degradation. Historically, factors like age, physical activity, and prevailing diseases have swayed protein dynamics. When this balance falters, with protein degradation gaining an upper hand, muscle atrophy, characterized by amplified protein degradation and subdued synthesis, ensues. The ubiquitin-proteasome pathway, a principal conduit for intracellular protein degradation, emerges as a common player in diverse skeletal muscle atrophy types. This article embarks on an exploration of the diverse manifestations of skeletal muscle atrophy. Grounded in this overview, we endeavor to decode and forecast the research trajectories surrounding the ubiquitin-proteasome pathway’s role in muscle atrophy processes, aiming to underpin future interventions and treatments in the realm of muscle disorders.

## 2 Protein synthesis and proteolysis in skeletal muscle atrophy

Skeletal muscle atrophy is characterized by a disrupted balance between protein synthesis and degradation, typically manifested by suppressed protein synthesis and/or enhanced protein degradation levels [1, 2]. Factors such as exercise and diseases commonly influence protein synthesis and degradation. Resistance training or anabolic hormonal stimuli can enhance protein synthesis, resulting in an increase in skeletal muscle cell protein synthesis, leading to an increase in muscle fiber size, a process known as skeletal muscle hypertrophy [3]. Under catabolic conditions, protein degradation surpasses synthesis, leading to muscle weakness and atrophy. Conditions such as disuse, sarcopenia, cancer-associated cachexia, malnutrition or starvation, and various other pathological states present with skeletal muscle atrophy [4].

Insulin-like growth factor-1 (IGF-1) is a pivotal growth factor in regulating the anabolic and catabolic pathways in skeletal muscles, with the phosphoinositide 3-kinase (PI3K)-AKT pathway considered the primary signaling protein cascade controlling muscle protein content [5]. Within skeletal muscle, the IGF-1-mediated PI3K/AKT pathway can enhance muscle protein synthesis by activating GSK3β and mTOR. The IGF-1/PI3K/AKT pathway suppresses protein degradation, especially of myofibrillar proteins, and inhibits the expression of the key atrophy gene Atrogin-1, thus increasing protein synthesis [3, 5]. One of the downstream targets of the protein synthesis pathway PI3K/AKT is the Forkhead box O (FOXO) transcription factor class. FOXO is a subfamily of the forkhead transcription factors. Mammalian cells contain three members of this family: Foxo1 (FKHR), Foxo3 (FKHRL1), and FOXO4 (AFX) [6]. Various studies suggest that the gene expression of Atrogin-1 and MuRF1 is differentially regulated by FOXO and NF-κB pathways [4, 7-9]. The IGF-1/PI3K/AKT pathway, by inhibiting FOXO, prevents the upregulation of Atrogin-1 and suppresses muscle atrophy, while FOXO activation alone can induce acute atrophy of myotubes and mature muscle fibers. FOXO3 activation is both a sufficient and necessary condition for rapid atrophy. FOXO3 is the primary transcription factor driving the expression of most atrophy genes, promoting overall protein hydrolysis [6, 10]. Atrogin-1 and MuRF1 are significantly upregulated by FOXO3 in all muscle atrophy scenarios [11-13].

During the atrophy process, the degradation of myofibrillar and soluble proteins accelerates, and protein synthesis also diminishes, resulting in a rapid decline in muscle mass and weight, escalating frailty and disability levels. This situation reduces the quality of life and can even lead to increased mortality [14]. With the rise in chronic disease patients and extended life expectancy, healthy aging and independent living require maintaining muscle mass [15]. Therefore, there is now a more pressing need than ever to deeply understand the molecular mechanisms governing muscle mass and function.

## 3 Effect of ubiquitin-proteasome pathway on skeletal muscle atrophy

Currently, four protein degradation pathways have been identified in skeletal muscle atrophy: the Ubiquitin-Proteasome Pathway (UPS), the autophagy-lysosome system, the calcium-dependent calpain system, and the cysteine aspartate protease system [16-20]. The UPS is responsible for the specific degradation of most intracellular proteins and serves as an efficient protein degradation route. In cells, the vast majority of proteins are degraded through the ubiquitin-proteasome system, hence it plays a crucial role in the skeletal muscle atrophy process [20].

The Ubiquitin-Proteasome Pathway consists of ubiquitin and a series of related enzymes that degrade intracellular proteins. It is mainly composed of three parts: ubiquitin, ubiquitin-related enzymes, and the proteasome [21]. Ubiquitin (Ub) is present in all eukaryotes (most eukaryotic cells) and is a small protein composed of 76 amino acids with a molecular weight of approximately 8,500. Its primary function is to tag proteins requiring degradation, allowing them to be degraded by the 26S proteasome [22]. Ubiquitin can be reused and ubiquitination can be reversed by removing ubiquitin from target proteins through deubiquitinating enzymes (DUBS) [23, 24]. Ubiquitin-related enzymes include E1 ubiquitin-activating enzyme, E2 ubiquitin-conjugating enzyme, E3 ubiquitin ligase, E4 ubiquitin chain extension enzyme, and deubiquitinating enzymes. Ubiquitin tagging of natively folded proteins is achieved through the first three enzymes. E3 ubiquitin ligase, a specific substrate-binding component, plays a pivotal role in the ubiquitin-mediated protein degradation cascade, determining the specificity and rate of UPS. The E3 ligase undergoes autoubiquitination, making it susceptible to proteasomal degradation, necessitating continuous transcriptional replenishment [3, 23]. The 26S proteasome, the primary protease of the ubiquitin-proteasome system, degrades polyubiquitinated proteins into peptides [25]. It consists of a 20S proteasome and one or two 19S proteasomes. These regulatory factors, when combined, enhance proteasome activity and allow the proteasome to degrade substrate proteins marked by complex enzyme machinery by attaching ubiquitin (Ub) molecule chains. The 20S can only recognize and degrade unfolded/damaged/misfolded proteins, while the 19S can recognize ubiquitinated proteins and other potential substrates of the proteasome, unfolding target proteins into protein chains to pass through the 20S core protein. Subsequently, substrates are degraded into short peptides, polyubiquitin chains are degraded into free monomers by deubiquitinating enzymes (DUB), and the released ubiquitin can be reused by the E1 enzyme [26]. Various types of E1, E2, and E3 enzymes facilitate the ubiquitination of different types of target proteins. The ubiquitination of proteins consists of four steps: 1) E1 activates ATP-dependent ubiquitin. 2) Activated ubiquitin is transferred to the E2 ubiquitin carrier protein. 3) E2 transfers the activated ubiquitin portion to a protein substrate specifically bound to E3. In the case of RING finger ligases, the transfer is direct, while in the case of HECT domain ligases, ubiquitin first forms an additional thioester intermediate on E3, then transferred from E3 to the substrate. 4) The continuous ligation of the ubiquitin portion produces polyubiquitin chains, which serve as a binding signal for the downstream 26S proteasome, causing the target substrate to degrade into peptides [21, 22, 27].

The ubiquitin-proteasome system involves nearly 750 E3 and about 90 DUB genes playing regulatory roles in skeletal muscle atrophy, referred to as atrogenes [28]. These atrogenes regulate various processes controlling muscle mass (such as myogenesis, protein synthesis, and degradation) and function in upstream regulatory pathways. These USP genes can be divided into three functions: 1) myofibril protein degradation, 2) myogenesis inhibition, and 3) autophagy regulation. During the ubiquitination process, E3 ligases are considered key enzymes catalyzing the activated form of Ub. Since their discovery in 2001, two muscle-specific E3 ubiquitin ligases, MuRF-1 and Atrogin-1 (MAFbx), have been shown to be associated with the regulation of skeletal muscle atrophy under various pathological and physiological conditions [29]. In mammalian muscles, MuRF1 and Atrogin-1 are responsible for the activation of protein degradation. Atrogin-1 and MuRF1 control the ubiquitination and subsequent forced degradation of regulatory proteins such as calcineurin phosphatase and MyoD as well as structural proteins like myosin and troponin I [3]. MuRF1 and Atrogin-1 are significantly upregulated during various types of atrophy. Additionally, inhibiting the expression of MuRF1 and Atrogin-1 can also partially counteract skeletal muscle atrophy [29-31].

### 3.1 The role of the ubiquitin-proteasome pathway in disuse muscle atrophy

The fundamental physiological principle of skeletal muscle is “use it or lose it.” Skeletal muscles exhibit significant plasticity, adapting according to functional demands. A reduction or cessation in activity levels typically leads to muscle atrophy and metabolic dysfunctions. The loss of skeletal muscle mass resulting from a sudden decrease in muscle loading and inhibited neural activation is often termed “disuse muscle atrophy.” Classic models of disuse muscle atrophy include bed rest, joint immobilization, mechanical ventilation, spaceflight, and denervation [32]. Any loss in skeletal muscle mass fundamentally stems from a chronic imbalance between muscle protein synthesis and degradation rates [33]. Muscle atrophy arises when the equilibrium between protein synthesis and degradation is disrupted, usually manifested by inhibited protein synthesis and elevated protein degradation, regardless of the duration of disuse. The loss in strength is often more than twice the muscle size reduction [34]. While there’s some debate about which of these factors predominates, most literature indicates that during the early stages of disuse (up to 10 days), protein synthesis remains relatively constant, while protein degradation increases considerably [35, 36]. That is, enhanced protein degradation is the primary cause of atrophy. For prolonged disuse (more than 10 days), the relationship with protein degradation becomes unclear, with decreased protein synthesis becoming the primary reason for muscle atrophy. The ubiquitin-proteasome system is generally considered the most crucial protein degradation system under disuse conditions. Under disuse, the ubiquitin-proteasome system is primarily regulated by the cellular energy sensor, AMP-activated protein kinase (AMPK). The role of AMPK in protein regulation evolves as the duration of disuse extends.

Studies have shown that when muscles are active, growth ceases; during muscle activity, the anabolic signaling pathway and protein synthesis are inhibited [37]. Eukaryotic elongation factor 2 (eEF2) and AMPK can lead to the suppression of the mTORC1/p70S6K signaling pathway and reduced mRNA translation efficiency [38, 39]. Upon cessation of activity, both anabolic and catabolic metabolism are activated to maintain intracellular homeostasis. From the first day of unloading, changes in adenosine monophosphate (AMP) concentration, accumulation of carbohydrates (CHO), and increased reactive oxygen species (ROS) concentration trigger a signaling cascade, resulting in the upregulation of E3 ubiquitin ligases. These factors culminate in alterations in protein metabolism and myosin phenotypes. On the first day of disuse, AMPK is dephosphorylated due to increased ATP accumulation in antigravity muscles compared to pectoral muscles, disrupting the ATP/AMP dynamic equilibrium [40-43]. Dephosphorylated AMPK might activate protein kinase D (PKD), with activated PKD further increasing AMPK dephosphorylation [41, 44]. Concurrently, CHO accumulation may also contribute to AMPK dephosphorylation [45]. As AMPK activity diminishes, cytoplasmic calcium concentration drops, and dephosphorylated eEF2 stimulates polypeptide chain elongation, activating the anabolic pathway, thus enhancing muscle protein synthesis rate [38, 39, 46]. However, with prolonged muscle disuse (e.g., 4–5 days), reduced mitochondrial oxidative phosphorylation leads to decreased ATP production. Subsequently, activated AMPK can phosphorylate six regulatory sites on FOXO3, promoting its nuclear retention and activation [47, 48], leading to the upregulation of key atrophy genes Atrogin-1 and MuRF1, resulting in muscle atrophy. For even more extended disuse periods (>10 days), research suggests that muscle atrophy is primarily due to decreased protein synthesis rates, without a significant increase in protein degradation [33]. Additionally, reactive oxygen species (ROS), crucial regulators in cellular signaling pathways, coupled with compromised antioxidant systems, are often regarded as major inducers affecting disuse muscle atrophy protein synthesis and degradation [49-51]. Intracellular Ca2+ overload, by activating caspase-3-dependent apoptosis, is also an essential signaling pathway in disuse muscle atrophy [52].

Current research on countering disuse muscle atrophy suggests that exercise is among the best strategies. Existing literature confirms that different types of resistance exercises enhance muscle protein synthesis by activating the PI3K-AKT-mTOR pathway, playing a crucial role in disuse models [53-55]. However, exercise might not be suitable for the elderly or those bedridden for extended periods, and during spaceflight, exercise can only partially delay microgravity-induced skeletal muscle atrophy [56]. Preliminary studies in our laboratory suggest that Rhodiola rosea can effectively counteract microgravity-induced muscle atrophy. Traditional Chinese herbs like Astragalus, Schisandra, and taurine have been proven in studies to reduce the expression of ubiquitin ligases Atrogin-1 or MuRF1 and inhibit disuse muscle atrophy [57-59], hinting at the potential of traditional medicine as a promising approach against disuse muscle atrophy in the future. Resveratrol, an antioxidant, has successfully counteracted disuse atrophy in studies [60]. Additionally, while protein supplementation combined with exercise yields optimal results, amino acid supplementation alone can notably improve protein synthesis but can only partially counteract muscle atrophy [61].

In conclusion, the primary cause of disuse muscle atrophy might be the skeletal muscle’s adaptive self-regulation in response to low energy consumption. Hence, mere energy supplementation might be the least effective remedy for disuse muscle atrophy; short-term disuse is mainly due to increased protein degradation, while long-term disuse relies on reduced protein synthesis. The combination of exercise and amino acid supplementation stands out as the best countermeasure for disuse muscle atrophy, with various traditional medicines and antioxidants demonstrating promising therapeutic potentials.

### 3.2 The role of the ubiquitin-proteasome pathway in sarcopenia

Sarcopenia is the age-related loss of skeletal muscle mass and function [62]. As age advances, numerous changes occur in skeletal muscle, such as the transformation of type II muscle fibers to type I, infiltration of fat within and between muscles, a decrease in the number of type II fiber satellite cells, and alterations in the integrity of mitochondria in muscle cells [63-67]. Aging disrupts the homeostasis of skeletal muscle, leading to an imbalance in the pathways of muscle protein synthesis and degradation, resulting in comprehensive muscle atrophy [68]. Concurrently, elderly individuals are prone to bedridden conditions due to illness, thus facing the dual challenges of disuse muscle atrophy and sarcopenia. A study by Landi and colleagues using the EWGSOP assessment method on individuals over 80 years old revealed that muscle loss syndrome is prevalent among the elderly (25%), and the risk of falls for these patients during a 2-year follow-up period is more than three times that of non-sarcopenic patients [69]. A study by Kitamura Akira indicated that the prevalence of sarcopenia among Asian men is 11.5%, and 16.7% among women [70]. Another study on sarcopenia suggested that the mortality risk for men depends on muscle mass, while for women, the risk is more influenced by low fat mass, muscle strength, and physical performance [71]. Given the growing aging population, combatting sarcopenia has become an urgent issue.

The 2018 International Clinical Practice Guidelines for Sarcopenia (ICFSR) highlighted that physical activity combined with resistance training can be the primary prescription for treating sarcopenia [72]. Steven’s research suggests that resistance and endurance exercises three times a week can significantly benefit sarcopenia by improving muscle mass, strength, and function. There are few exercise experiments designed specifically for sarcopenia patients, and the methods, durations, and intensities vary, but all have demonstrated the effectiveness of exercise against sarcopenia [73-75]. Some evidence suggests that adequate intake of protein, vitamin D, antioxidants, and long-chain polyunsaturated fatty acids is beneficial for health [76], but their individual effects on sarcopenia have not been reported. Nevertheless, protein and vitamin D supplements remain essential components for sarcopenia treatment. Alfonso reported that essential amino acid (EAA) leucine and β-hydroxy-β-methylbutyrate (HMB) supplements have a role in improving muscle mass and function [77]. Currently, no specific drugs have been approved for sarcopenia. Aging is accompanied by a reduction in testosterone and growth hormone secretion. A report by Manthos revealed that the combined administration of testosterone and growth hormone is more effective than either hormone alone [78]. A recent study suggested that inhibiting muscle growth inhibitors might be a new direction to combat sarcopenia [79].

Early studies found that the ubiquitin levels in the extensor digitorum longus (EDL) of elderly rats (24 months old) and the quadriceps of humans aged 70–79 years were significantly increased compared to normal levels [80]. Recently, some researchers detected that the overall ubiquitin level in rat bicep femoris muscle increases with age [81]. However, contradicting findings have emerged. A study by Leslie showed no difference in the overall ubiquitin levels in the bicep femoris muscles of 9-month-old and 29-month-old male rats[^82^]. The role of Atrogin-1 and MuRF1 in sarcopenia research has shown conflicting results. In Clavel’s report, the mRNA levels of Atrogin-1 and MuRF1 in the tibialis anterior (TA) muscle of 24-month-old rats significantly increased compared to 5-month-old controls [83]. Other studies reported either unchanged or decreased levels of Atrogin-1 and MuRF1 [82, 84]. Darren and colleagues found that knocking out the MuRF1 gene in 24-month-old mice resulted in a significant reduction in muscle weight, strength, and cross-sectional area (CSA) compared to controls[85]. Some found that the lifespan of Atrogin-1 knockout mice is shorter than normal mice, and they experience higher muscle mass loss during aging [86], suggesting that E3 ubiquitin ligases play a crucial role in maintaining muscle mass during mouse aging.

In summary, in studies related to age-associated skeletal muscle atrophy, both exercise and hormonal or drug therapies have demonstrated effectiveness against atrophy. However, research on their development and combat mechanisms, especially concerning the ubiquitin pathway, is limited, and findings have been inconsistent. This illustrates the complexity of the causes of sarcopenia and suggests a need for further research to clarify the mechanisms underlying the onset of sarcopenia.

### 3.3 The role of the ubiquitin-proteasome pathway in cancer cachexia

Cancer Cachexia (CaCax), often simply referred to as cachexia, is a severe complication of cancer. Approximately one-third of cancer-related deaths can be attributed to cachexia [87]. It is characterized by progressive atrophy of skeletal muscles and adipose tissues, weight loss, decline in quality of life, and reduced life expectancy [88, 89]. Studies have indicated that cachexia cannot be prevented or alleviated by mere dietary changes or nutritional augmentation [90]. Multiple experiments have demonstrated that inhibiting skeletal muscle atrophy in cachexia patients can significantly prolong their lifespan [91, 92]. Thus, the prevention and treatment of cachexia might be an essential prerequisite for treating cancer patients.

Distinct from other types of muscle atrophy, a hallmark of cancer cachexia is pervasive systemic inflammation [93, 94]. Inflammatory cytokines regulate the intracellular signaling pathways associated with muscle atrophy. *In vitro* studies suggest that these cytokines can elevate the expression of muscle-specific ubiquitin ligases, Atrogin-1 and MuRF1. Pro-inflammatory cytokines partly induce muscle atrophy by modulating the AKT/FOXO/UPS pathway. Phosphorylation of AKT inhibits protein catabolism transcription factors. The full activation of AKT, which promotes protein synthesis and induces FOXO1, initially accelerates protein degradation and contributes to MuRF1 and Atrogin-1 transcription during muscle atrophy. Pro-inflammatory cytokine Interleukin-6 (IL-6) is considered a central mediator of cancer cachexia, with IL-6 levels correlating positively with the progression of cachexia [95]. Baltgalvis et al. found that in cancer mice, systemic concentrations of IL-6 increased with tumor progression and correlated with elevated p-STAT-3 and Atrogin-1 mRNA levels [96]. Research indicates that IL-6 binds with the IL-6r-Gp130 receptor complex and activates the JAK tyrosine kinase. Conformational changes in JAK proteins activate the STAT proteins through phosphorylation [97]. STAT transcriptional activation induces muscle atrophy through various mechanisms. STAT stimulates the activity of CCAAT/enhancer-binding protein (C/EBPδ), thereby enhancing the expression of myostatin, Atrogin-1, MuRF1, and caspase-3 [98, 99]. Furthermore, in cachexia-induced muscle atrophy, inflammation can suppress IGF-1, leading to enhanced catabolism [100]. IGF-1 is a pivotal growth factor regulating both the anabolic and catabolic pathways of skeletal muscle. Inhibiting the IGF-1/PI3K/AKT pathway results in protein degradation, particularly of myofibrillar proteins, and an increased expression of Atrogin-1 [3, 5, 101]. Even though inflammation has been proven to play a crucial role in the development of cachexia, more insight is needed into how these cytokines advance disease progression to either prevent or delay cachexia’s onset.

In conclusion, substantial evidence from both animal and human studies suggests that the UPS is activated during skeletal muscle atrophy in cancer cachexia. Muscle-specific E3 ubiquitin ligases MuRF1 and Atrogin-1 are notably upregulated during the onset and progression of cancer cachexia, with inflammation playing a pivotal role in the development of muscle atrophy in cachexia. Due to the complex etiology of diseases like cancer, no effective measures have been identified to counteract the muscle atrophy they induce. Hence, there’s a pressing need for further research into the role of UPS in skeletal muscle atrophy during cancer cachexia in humans.

## 4 Summary and outlook

Historically, the physiological challenge posed by skeletal muscle atrophy has been of paramount importance. Its implications span across the potential extension of human lifespan, advances in cancer treatment, and even the bold aspirations of interstellar exploration. The struggle against skeletal muscle atrophy has, and continues to be, a pressing matter in the annals of scientific inquiry.

Contemporary research delineates that while every decrement in skeletal muscle mass is invariably anchored in a persistent disequilibrium between muscle protein synthesis and degradation, muscle atrophy, dependent on its etiology, manifests distinct pathophysiological characteristics. Disuse muscle atrophy, for instance, can largely be ascribed to metabolic adaptations to diminished energy demands, and interventions such as resistance training coupled with amino acid supplementation emerge as primary counteractive measures. In the realm of age-induced muscle atrophy, modalities ranging from exercise regimens to hormonal therapeutics have been spotlighted for their potential therapeutic benefits. When navigating the complexities of cancer cachexia, the omnipresent shadow of inflammation looms large, underpinning muscle wasting processes. Despite cachexia’s multifarious etiological landscape, therapeutically targeting inflammation presents itself as an imperative.

Yet, within the corpus of extant literature, the exploration of the ubiquitin-proteasome system (UPS) in skeletal muscle atrophy remains, surprisingly, in its nascent stages. Elevated expressions of diverse E3 ligases have been identified across various atrophic conditions. Inhibition of the UPS pathway offers a tantalizing prospect for mitigating muscle wasting. However, a mosaic of experimental results has emerged, with some indicating that certain E3 gene knockouts neither halt nor alleviate muscle atrophy, and might even accentuate its progression. Thus, the labyrinth of UPS in skeletal muscle atrophy’s mechanistic underpinnings is yet to be fully navigated. Seminal studies, notably those centered around MuRF1 and Atrogin-1, pave the way, but the journey ahead mandates a deeper scholarly quest to unveil precise mechanisms, thereby fortifying our theoretical armamentarium for muscle atrophy’s prevention and treatment.
